# Extracellular Vesicle miRNAs in the Promotion of Cardiac Neovascularisation

**DOI:** 10.3389/fphys.2020.579892

**Published:** 2020-09-25

**Authors:** Despoina Kesidou, Paula A. da Costa Martins, Leon J. de Windt, Mairi Brittan, Abdelaziz Beqqali, Andrew Howard Baker

**Affiliations:** ^1^Centre for Cardiovascular Science, The Queen's Medical Research Institute, The University of Edinburgh, Edinburgh, United Kingdom; ^2^Department of Molecular Genetics, Faculty of Science and Engineering, Maastricht University, Maastricht, Netherlands; ^3^Faculty of Health, Medicine and Life Sciences, Cardiovascular Research Institute Maastricht, Maastricht University, Maastricht, Netherlands

**Keywords:** extracellular vesicles (EV), microRNA (miR), neovascularisation, angiogenesis, cardiac, myocardial infarct, exosome (EXO), regeneration

## Abstract

Cardiovascular disease (CVD) is the leading cause of mortality worldwide claiming almost 17. 9 million deaths annually. A primary cause is atherosclerosis within the coronary arteries, which restricts blood flow to the heart muscle resulting in myocardial infarction (MI) and cardiac cell death. Despite substantial progress in the management of coronary heart disease (CHD), there is still a significant number of patients developing chronic heart failure post-MI. Recent research has been focused on promoting neovascularisation post-MI with the ultimate goal being to reduce the extent of injury and improve function in the failing myocardium. Cardiac cell transplantation studies in pre-clinical models have shown improvement in cardiac function; nonetheless, poor retention of the cells has indicated a paracrine mechanism for the observed improvement. Cell communication in a paracrine manner is controlled by various mechanisms, including extracellular vesicles (EVs). EVs have emerged as novel regulators of intercellular communication, by transferring molecules able to influence molecular pathways in the recipient cell. Several studies have demonstrated the ability of EVs to stimulate angiogenesis by transferring microRNA (miRNA, miR) molecules to endothelial cells (ECs). In this review, we describe the process of neovascularisation and current developments in modulating neovascularisation in the heart using miRNAs and EV-bound miRNAs. Furthermore, we critically evaluate methods used in cell culture, EV isolation and administration.

## Introduction

CVD is the leading cause of mortality worldwide with the World Health Organization (WHO) reporting that in 2016, 17.9 million people died from CVDs. Of those deaths, an estimated 7.4 million were attributed to CHD alone (WHO Cardiovascular diseases CVDs, 2017). CHD is characterised by narrowing of the coronary arteries due to the gradual formation and the subsequent rupture of plaque within the vessel walls. Blockage of these arteries results in oxygen and nutrient deprivation of the downstream tissue. Consequently, ischaemic damage and cardiomyocyte death occur in the affected region of the heart, a phenomenon known as MI (Thygesen et al., 2019). Various methods, including stem cell (SC) injection, growth factor (GF) delivery, and miRNA treatment, have been proposed to reduce cardiomyocyte death; nonetheless, several limitations have restricted their use. The idea of restoring the lost myocardium was proven to be inefficient since adult mammal cardiomyocytes are terminally differentiated and in contrast to other vertebrate species, such as zebrafish, adult mammal cardiac regenerative capacity is limited (Ye et al., 2018). For this reason, SC injection was proposed as a promising strategy for cardiac repair. The initial hypothesis was that transplanted SCs would differentiate into cardiomyocytes, integrate into the host myocardium, and augment cardiac function through synchronised electromechanical coupling (Garbern and Lee, 2013; Lemcke et al., 2018). Despite some encouraging results, this approach has proven to be largely unsuccessful to date due to several issues, including poor engraftment, immune rejection, genetic instability, and possible teratocarcinoma formation. Importantly, the engrafted cells have also demonstrated relatively poor electrical coupling with the host myocardium, leading to the development of additional safety issues (Liu et al., 2018; Menasché et al., 2018; Romagnuolo et al., 2019). Recently, Vagnozzi et al. (2020), showed that the improvement in cardiac function following SC transplantation may be due to an acute inflammatory wound-healing response, rather than incorporation of delivered cells or production of new cardiomyocytes.

Aiming to improve recovery after MI and, therefore, rescue as many cardiomyocytes as possible, research interest is focused on promoting neovascularisation to create new myocardial blood vessel networks. Paracrine cell communication plays a critical role in the control of this process (Gnecchi et al., 2008; Mirotsou et al., 2011; Hodgkinson et al., 2016). Cell communication in a paracrine manner is regulated by several mechanisms, including EVs. EVs carry and transfer various bioactive molecules, such as small non-coding RNAs, proteins, and lipids, that moderate signalling pathways in the recipient cells. These functional contents depend on several factors, including the cellular origin and the (patho)physiological state of the cells during EV packing and secretion (Raposo and Stoorvogel, 2013). Recently, EV-miRNAs have gained immense interest in the control of cell behaviour in the recipient cells. miRNAs are small non-coding RNAs of approximately 22 nucleotides and have been recognised as critical regulators of gene expression. While most miRNAs regulate gene expression by binding to the 3′ untranslated region of their messenger RNA (mRNA) target, there are also sporadic reports of miRNAs that inhibit protein translation by binding to the 5′ untranslated region of their target. Under certain conditions, miRNAs can also control transcription or activate translation (O'Brien et al., 2018). Nonetheless, there is great controversy regarding the functional relevance of miRNAs in EVs. Several reports suggest that the majority of extracellular miRNAs are protected from plasma ribonucleases by forming complexes with proteins, such as Ago2 and that only a few copies of each miRNA are found in EVs (Arroyo et al., 2011; Chevillet et al., 2014). Moreover, in a non-peer-reviewed study, it was recently argued that miRNAs are not effectively delivered to target cells in a functional manner (Albanese et al., 2020). On the other hand, numerous studies have demonstrated that protected from plasma ribonucleases by their EV carriers, functional miRNAs can be delivered and internalised into recipient cells, acting as novel regulators of gene expression by inhibiting their targets (Johnson et al., 2019; Qiao et al., 2019; Cavallari et al., 2020). Despite this controversy, preclinical studies have demonstrated that EVs hold promise in the regulation of complex processes such as neovascularisation. In this review, we describe the process of neovascularisation and current developments in modulating neovascularisation in the heart using miRNAs and EV-bound miRNAs. Furthermore, we critically evaluate methods used in cell culture, EV isolation and routes of EV administration.

## Post-ischaemic Neovascularisation

After the onset of myocardial ischaemia due to coronary artery occlusion, there is an inadequate blood supply to the heart muscle, which results in a pathological change in electrical, contractile or biochemical function of the heart (Grover, 1995). Therefore, post-ischaemic neovascularisation is essential to support the metabolic needs of cardiac cell populations. Angiogenesis and arteriogenesis are regulated by distinct, but partially overlapping, cellular and molecular mechanisms and are responsible for tissue repair and remodelling in acute and chronic ischaemic diseases (Persson and Buschmann, 2011). Angiogenesis refers to the formation of new blood vessels from pre-existing vessels and can be classified as intussusceptive or sprouting angiogenesis (Caduff et al., 1986; Burri and Tarek, 1990; Risau, 1997). Intussusceptive angiogenesis is a dynamic splitting process in which elements of interstitial tissues invade existing blood vessels forming a cylindrical microstructure that spans the lumen of capillaries and small vessels (Short, 1950; Caduff et al., 1986).

**Figure 1:**
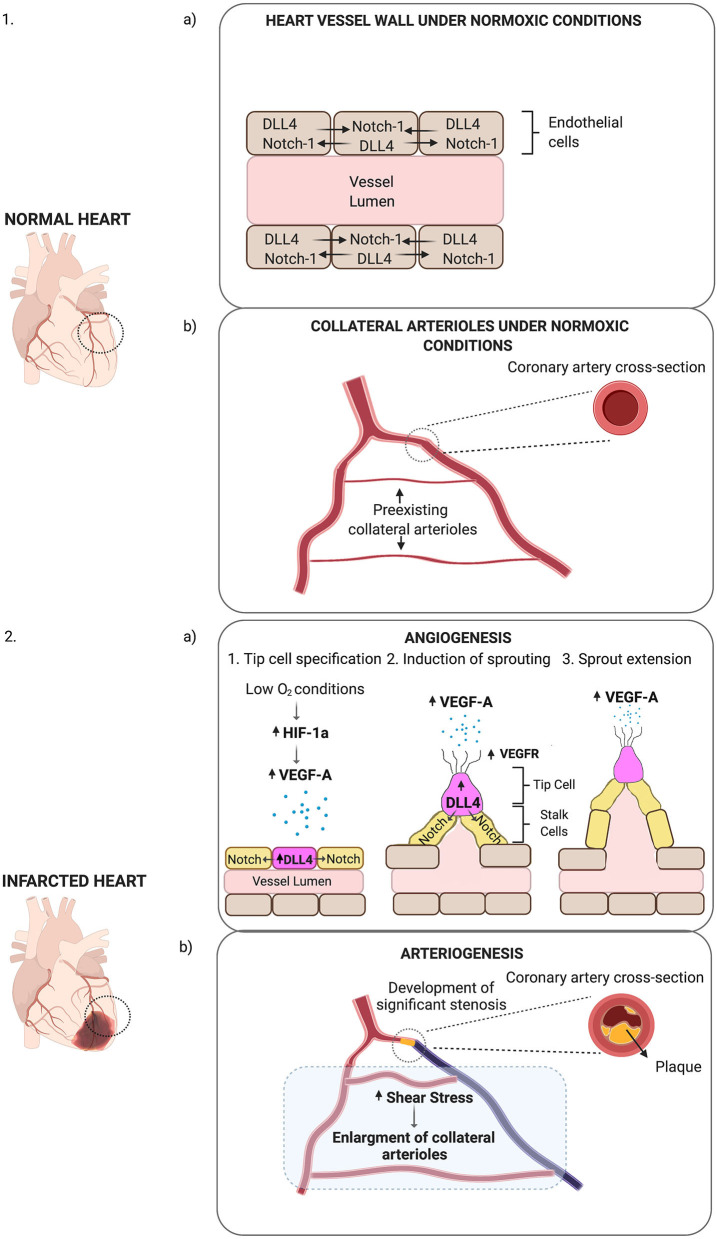
Schematic representation of post-ischaemic neovascularisation. **(1a)** Under normoxic conditions, DLL4-Notch-1 signalling is balanced in ECs. **(1b)** Under normoxic conditions, small collateral arterioles may connect main arteries. **(2a)** Low O_2_ conditions increase HIF-1a levels, which in turn stimulates the expression of VEGF-A (represented as small blue dots). In response to VEGF-A, Dll4 expression increases on the surface of tip cells (purple cell). Dll4 receptors on tip cells bind to Notch receptors on the surface of stalk cells (yellow cells) activating Dll4/Notch signalling pathway. Dll4—Notch-1 signalling directs ECs into migrating tip or proliferating stalk cells. In response to VEGF-A, VEGFR expression increases on the filopodia of tip cells (represented as protrusions on the tip cell), which then migrate towards VEGF-A with the stalk cells proliferating behind them. This results in the expansion of the vessel lumen and the formation of sprouts on the vessel wall (angiogenesis). **(2b)** When a coronary artery is occluded due to the formation of plaque within the arterial wall, there is increased shear stress in the collateral arterioles. In response to shear stress, collateral arteriole diameter increases in size (arteriogenesis).

As implied by its name, sprouting angiogenesis is characterised by EC sprouts, which usually grow toward an angiogenic stimulus. Hypoxia is one of the key drivers of this process. The primary mechanism of hypoxia-induced neovascularisation involves an increase in hypoxia-inducible factor (HIF-1a) levels (Figure 1). When oxygen-sensing mechanisms detect low oxygen levels, new blood vessels are required to satisfy the metabolic requirements of parenchymal cells. Most of these cells respond to hypoxic stimuli by secreting a critical proangiogenic growth factor, vascular endothelial growth factor (VEGF-A). A particular type of EC, known as tip cells, leads sprouting angiogenesis (Gerhardt et al., 2003). The filopodia of endothelial tip cells are endowed with VEGF-A receptors (VEGFR2) that allow them to sense changes in VEGF-A concentrations and drive them to align with the VEGF gradient. Neighbouring cells, known as stalk cells, divide as they follow behind a tip cell, elongating the new vessel. Maturation and stabilisation of the vessels involve the recruitment of pericytes and ECM deposition along with mechanical stimuli (Stratman and Davis, 2012). This process is highly conserved and regulated by very specific molecular pathways, such as the Notch signalling pathway. In response to VEGF-A, the expression of the ligand Dll4 increases on the surface of tip cells. As a result, these ligands bind to Notch1 receptors of adjacent cells and activate Notch signalling pathway that suppresses tip cell fate and induces the stalk cell phenotype in these cells (Del Toro et al., 2010).

While angiogenesis is stimulated by low-oxygen concentrations and leads to the creation of new capillaries, arteriogenesis is induced by physical forces; mainly fluid shear stress. Arteriogenesis is an adaptive response to transient, repetitive occlusion of a large main artery and is characterised by an enlargement of the collateral arterioles (Figure 1). Once a main artery is occluded the arterial pressure behind the stenosis site decreases, and the blood is redistributed via the collateral arterioles. Thus, collateral arterioles now connect a high-pressure with a low-pressure region (Schaper and Pasyk, 1976). While hypoxia-induced pathways, as well as alterations in haemodynamic forces of the vascular wall, have been proposed as critical regulators of vessel growth after an ischaemic event, emerging evidence supports the idea that tissue-resident EPCs may also be involved in cardiac neovascularisation post-ischaemia (Fujisawa et al., 2019). A better understanding of the cellular frameworks and molecular mechanisms that drive these processes is crucial for the development of new therapeutic neovascularisation strategies.

The use of animal models is essential for a better understanding of the neovascularisation process post-MI at a cellular level. Aiming to identify the mechanism of EC proliferation post-ischaemia, Manavski et al. (2018), utilised Confetti fl/wild type (wt) cadherin 5 (Cdh5)- CreER^T2^ mice and revealed that ischaemia-induced vascularisation post-acute myocardial infarction (AMI) is mediated by clonally expanded ECs. At day 7 post-AMI 28% of the cardiac sections showed significant clonal expansion of Cdh5-expressing ECs, and this was increased to 33% on day 14. Since Cdh5 is also expressed in bone marrow, the origin of these clonally expanded cells remained inconclusive. Recently, Li et al. (2019) utilised a Pdgfb-iCreER -R26R-Brainbow2.1 mouse. In this system, the expression of *Cre* is driven by a Pdgfb promoter, which is specific to ECs (Claxton et al., 2008). Using this mouse, they demonstrated that vessel formation and clonal expansion of cardiac ECs was mediated by a subpopulation of resident cardiac ECs with progenitor-like properties.

Genetic lineage tracing has significantly improved our understanding of the neovascularisation process in the post-ischaemic heart. Nonetheless, cardiac neovascularisation potential is limited and does not appear to effectively promote myocardial regeneration. Recently, Kocijan et al. (2020), used an Apln-CreER;R26mT/mG mouse model to compare the angiogenic potential of the heart and skeletal muscle. Apln is highly expressed in ECs during embryonic development and is down-regulated in adulthood. However, in response to hypoxia, under tissue ischaemia or in the context of a tumour, the expression of Apln is reactivated, particularly in tip cells. Using this system, the authors showed that different pro-angiogenic stimuli activated Apln in skeletal muscle, resulting in angiogenic sprouts that could be incorporated into arteries. In the heart, however, Apln+ cells failed to give rise to new vessels. To confirm these data, the authors implanted cancer cells in different organs and showed that the angiogenic response in the heart was reduced. These data confirm that the inherent angiogenic response of the cardiac muscle is limited, emphasising the need for new therapeutic approaches to promote endogenous neovascularisation.

### MicroRNAs in Therapeutic Neovascularisation

Over the past few years, miRNAs have gained widespread attention for their role in vascular health and disease, including in neovascularisation. MiRNAs are small (18–22 nucleotide, nt) endogenous non-coding RNA molecules that negatively regulate gene expression by targeting specific mRNAs. Most target sites on mRNAs only share a partial complementarity with their corresponding miRNAs, and thus, a single miRNA can target multiple mRNAs, contributing to biological and pathophysiological processes (Huntzinger and Izaurralde, 2011).

Emerging evidence suggests that miRNAs are critical regulators of both adaptive and maladaptive vascular remodelling and angiogenesis. Table 1 contains a list of all known miRNAs that play a role in cardiovascular neovascularisation as well as their experimentally confirmed targets. Some of these have been extensively studied. MiR-126, for instance, is one of the most abundantly expressed miRNAs in ECs and has a prominent role in controlling angiogenesis by repressing negative regulators of the VEGF pathway, such as the Sprouty-related protein SPRED1 and phosphoinositol-3 kinase regulatory subunit 2 (PIK3R2/p85-beta) (Fish et al., 2008; Wang et al., 2008; Schober et al., 2014). Wang et al. (2008), showed that targeted deletion of miR-126 in mice leads to leaky vessels, haemorrhage and embryonic lethality due to defective vascular integrity. Half of the animals survived 1 week post-MI, while almost all died within 3 weeks post-MI. Another miRNA with angiogenic properties is miR-210. MiR-210 upregulation is a principal element of EC response to hypoxia (Fasanaro et al., 2009). Hu et al. (2010), demonstrated that overexpression of miRNA-210 post-MI in mice increased post-ischaemic neovascularisation by inhibiting ephrin-A3 and improved cardiac function 8 weeks post-MI. miR-23-24-27 cluster has also been reported to play a critical role in the regulation of neovascularisation. Knock-out of miR-27b, a component of this cluster impaired capillary branching in zebrafish embryos by targeting Dll4 and Sprouty (Spry)-2 (Biyashev et al., 2012). Veliceasa et al. (2015), also showed that overexpression of miR-27b in a mouse MI model increased capillary density and reperfusion, and improved cardiac function with an approximately 2-fold increase in ejection fraction over the control 14 days post-MI, and significantly reduced fibrosis at day 28.

**Table 1:** miRNAs playing a role in cardiovascular neovascularisation.

**miRNA**	**Model**	**Effect**	**Experimentally confirmed molecular targets**	**References**
**miRNAs THAT IMPROVE NEOVASCULARISATION**				
miR-10a/10b	miR-10a and miR-10b deficient & WT zebrafish embryos	Impaired blood vessel outgrowth in miR-10a and miR-10b deficient zebrafish embryos compared with controls	mindbomb E3 ubiquitin protein ligase 1 (mib1)	Wang et al., 2016
miR-21	Cardiac microvascular endothelial cells (CMVECs) Sprague-Dawley rats -MI induction by LAD coronary artery ligation Chicken chorioallantoic membrane (CAM)	Increased EC tube formation, proliferation and decreased apoptosis post-miR21 overexpression *in-vitro*. Improved cardiac function post-miR-21 overexpression in rats. Opposite effects post-miR inhibition *in-vivo*. Implantation of matrigel plugs containing miR-21-overexpressing DU145 cancer cells onto the CAM of a chicken embryo resulted in increased microvessel formation compared to controls	Phosphatase and tensin homolog (PTEN)	Liu et al., 2011; Yang et al., 2016
miR-26b	HUVECs Male CD-1 mice-HLI induction post-left femoral artery ligation	Increased EC proliferation, migration and tube formation post-miR overexpression *in-vitro*. Decreased EC proliferation and tube formation post-miR-inhibition *in-vitro*. Increased microvessel formation in a Matrigel plug model. Overexpression of miR-26b in a mouse HLI model improved capillary survival in the ischaemic muscles	PTEN	Martello et al., 2018
miR-23-24-27 cluster *(miR-23a/b, miR-27a/b, miR-27a-3p)*	HUVECs EC spheroids miR-27b knock-out & WT zebrafish embryos aortic rings from athymic nude mice FVB mice-HLI induction C57/Bl6 mice- MI induction by LAD coronary artery ligation	Decreased EC sprouting in aortic rings *ex-vivo* post-miR-27b knock-down. Impaired capillary branching in miR-27b knock-out zebrafish embryos compared with controls. Increased capillary density and reperfusion post-miR-27b injection in mouse HLI and MI models compared with controls. Inhibition of miR-23a/b and miR-27a/b in HUVECs significantly impaired their tube formation ability. Aortic ring treatment with anti-miR-23 or anti-miR-27 resulted in impaired EC sprouting. Opposite results post miR-23b and miR-27b overexpression. Overexpression of miR-27a-3p in HUVECs significantly increased tube formation. Opposite results post-miRNA inhibition. Overexpression of miR-27a or miR-27b in EC spheroids, resulted in significantly increased EC sprouting compared to controls. MiR-27a/b inhibition led to opposite results.	Spry-2 (target of both miR-23 and miR-27) Dll4 (target of miR-27b) Semaphorin 6A (SEMA6A) (target of both miR-23 and miR-27)	Zhou et al., 2011; Biyashev et al., 2012; Veliceasa et al., 2015; Rao et al., 2019
miR-29a	HUVECs	Increased EC tube formation and proliferation post-miR overexpression *in-vitro*. Opposite effects were observed post-miR inhibition in EC *in-vitro*	HMG-Box Transcription Factor 1 (HBP1)	Yang et al., 2013
miR-30a	Transgenic (Tg) friend leukaemia integration 1 transcription factor (fli1): enhanced green fluorescent protein (EGFP) zebrafish	MiR-30a loss of function decreased arteriolar sprouts compared to controls. miR-30a gain of function increased arteriolar branching	Dll4	Jiang et al., 2013
miR-31 miR-720	EPCs from patients with CHD nude mice- HLI induction by ligation of both proximal and distal portion of the right femoral artery, as well as the distal portion of saphenous artery	Increased tube formation and migration of EPC post-miR-31/−720 overexpression. Opposite effects were observed post-miR inhibition. Increased blood flow by intramuscular injection of EPCs overexpressing miR-31	Thromboxane A2 receptor (miR-31 target) Vasohibin-1 (miR-720 target)	Wang et al., 2014
miR-101	HUVECs Aortic rings from female C57BL/6J mice Female C57Bl/6J mice- HLI induction by double ligation of the superficial femoral artery proximal to the deep femoral artery and distal femoral artery	Increased EC tube formation, proliferation and migration *in-vitro* post-miR-101 overexpression *in-vitro*. Increased EC sprouting *ex-vivo* post-transfection with mir-101-expressing lentivirus. Increased capillary density and limb perfusion in miR-101 injected mice compared with controls	CUL 3	Kim et al., 2014
miR-106b-93-25 cluster *(miR-106b)*	Primary bone marrow stromal cells (BMSCs) from Female WT & miR-106b~25 knock-out mice Aortic rings from mir-106b~25 wild-type or knock-out mice Female WT & miR-106b~25 KO mice—HLI induction by femoral artery ligation	Increased tube formation ability and survival of BMSCs from WT compared to BMSCs from miR-106b~2 knock-out mice. Increased capillary sprouting in WT compared to miR-106b~2 knock-out mice. Significantly increased blood flow and number of lectin-positive capillaries in the WT compared with the KO mice on Day 7 & Day 14 post-ischaemia	Unknown	Semo, 2013
miR-126	Human aortic endothelial cells (HAECs) HUVEC Aortic rings from miR-126^−/−^ mice Male NMRI nude mice- MI induced by LAD coronary artery ligation miR-126^−/−^ mice- MI induced by LAD coronary artery ligation	Improved HAEC tube formation post-miR-126 transfection *in-vitro*. Opposite effects post-miR inhibition *in-vitro*. Improved HUVEC tube formation post-miR-126-expressing adenovirus (Ad-miR-126) transfection *in-vitro*. Opposite effects were observed post-miR inhibition *in-vitro*. Impaired EC sprouting in aortic rings from miR-126^−/−^ mice *ex-vivo*. Improved cardiac function and capillary density post-miR-126 mimic injection in NMRI nude mice post-MI. Opposite effects post-miR inhibition *in-vivo*. Reduced survival of miR-126^−/−^ mice post-MI (half of the mice died 1week post-MI, and nearly all of them died within 3 weeks post-MI)	SPRED1 PIK3R2	Fish et al., 2008; Wang et al., 2008; Jakob et al., 2012
miR-130a	HUVECs	HUVEC co-transfection with pcDNA3.1-miR-130a and pcDNA3.1-growth-arrest-homeobox-transcription-factor (GAX) showed that miR-130a antagonised GAX-induced inhibition of HUVEC tube formation and migration, increasing tube formation and migration *in-vitro*. miR-130a inhibitor reversed antagonism of GAX activity	GAX	Chen and Gorski, 2008
miR-132	HUVECs	Increased HUVEC tube formation ability and proliferation post-miR overexpression. Opposite effects were observed post-miR inhibition in EC *in-vitro*	p120 Ras GTPase activating protein (p120RasGAP)	Anand et al., 2010
miR-150	HUVECs exposed to oxidised low-density lipoprotein (LDL) Apolipoprotein E-deficient (ApoE^−/−^) mice-HLI induction by femoral artery ligation	Overexpression of miR-150 rescued the decreased tube formation ability of HUVECs exposed to LDL. Intramuscular injection of miR-150 mimic increased capillary and arteriolar (arteriogenesis) densities compared with controls	SRC Kinase Signalling Inhibitor 1(SRCIN1) (previously identified target)	Desjarlais et al., 2017
miR-210	Human umbilical vein endothelial cells (HUVECs), Aortic rings from female C57BL/6 mice, miR-210 overexpressing & WT mice subjected to cardiac ischaemia/reperfusion or permanent LAD coronary artery ligation	Increased HUVEC tube formation post-miR210 overexpression *in-vitro*. Opposite effects were observed post-miR inhibition *in-vitro*. Increased EC sprouting in aortic rings from miR-210 overexpressing mouse hearts *ex-vivo*. Increased reperfusion, capillary density and improved cardiac function in miR-210 overexpressing mice compared with controls both after ischaemia-reperfusion and MI induction	Ephrin-A3	Hu et al., 2010; Arif et al., 2017
14q32 cluster *(miR-329, miR-487b, miR-494, and miR-495)*	Male C57BI/6 mice-HLI induction by electroagulation of the left femoral artery proximal to the superficial epigastric arteries (single ligation-model for effective arteriogenesis), or by electroagulation of the distal femoral artery proximal to the bifurcation of the popliteal and saphenous artery (double ligation-model for severe peripheral arterial disease)	Silencing of miRNAs was induced by gene silencing oligonucleotides (GSO). Treatment with all 4 GSO improved blood flow recovery post-ischaemia *in-vivo*. GSO-495 and GSO-329 treatment increased perfusion 3 days post-ischaemia. Treatment with GSO-329 only, increased perfusion 7 days post-ischaemia. Increased collateral artery diameters (arteriogenesis) and capillary densities post-GSO treatment in mice compared with controls	Myocyte enhancer factor 2A (MEF2a) (target of miR-329) FGFR2, VEGF-A, ephrin-2 (targets of miR-494)	Welten et al., 2014
miR-424	HUVECs	Increased tube formation, migration and proliferation of EC *in-vitro* post-miRNA overexpression. Opposite effects were observed post-miR inhibition in EC *in-vitro*	Cullin 2 (CUL2)	Ghosh et al., 2010
miR-503	HUVECs Human microvascular endothelial cells (HMVECs)	Increased tube formation and migration of EC *in-vitro* post-miRNA overexpression. Opposite results were observed post-miR inhibition in EC *in-vitro*	Cyclin E1 (CCNE1) Cell division cycle 25 A (cdc25A)	Caporali et al., 2011
let-7f	HUVECs	Decreased tube formation and migration of EC *in-vitro* post-miRNA inhibition	Unknown	Kuehbacher et al., 2007
**miRNAs THAT INHIBIT NEOVASCULARISATION**				
miR-15a/ miR-16-1 cluster *(miR-15a, miR-16)*	HUVECs EC-selective MiR-15a Transgenic (EC-miR-15a TG) & WT mice- HLI induction by femoral artery ligation Male CD1 mice- LI induction by left femoral artery ligation	Increased HUVEC tube formation and migration post-miR inhibition *in-vitro*. Opposite results post-miR-overexpression *in-vitro*. Decreased number of capillaries and blood perfusion in EC-miR-15a TG mice compared with controls. Inhibition of miR-15a/16 in mice post HLI improved capillary density and blood perfusion	FGF2 VEGF	Yin et al., 2012; Besnier et al., 2019
miR-15b-5p	HUVEC Male C57BL/6 mice- HLI induction by left femoral artery ligation	Decreased HUVEC tube proliferation and migration post-miR overexpression *in-vitro*. Decreased blood flow recovery, capillary and arterial density in HLI mice post-miR-15b-5p overexpression	AKT Serine/Threonine Kinase 3 (AKT3)	Zhu et al., 2017
miR-16 miR-426	HUVECs	Decreased EC tube formation and migration post-miR overexpression *in-vitro*	VEGF receptor-2 (VEGFR2) (target of both miRNAs) Fibroblast growth factor receptor-1 (FGFR1) (target of both miRNAs)	Chamorro-Jorganes et al., 2011
miR-22	Fluorescent-labelled transgenic zebrafish Tg(fli1:EGFP) embryo expressing GFP in ECs	MiR-22 injection in zebrafish embryos resulted in defective vascular development which was rescued with miR-22 and VE-cadherin co-injection. miR-22 negative control (NC) injection in zebrafish embryos resulted in normal vessel development	Vascular endothelial (VE)-cdh	Gu et al., 2017
miR-23-24-27 cluster *(miR-24)*	HUVEC Tg(kdrl:eGFP)s843 zebrafish embryos Male C57BL/6 mice- MI induction by LAD coronary artery ligation	Decreased HUVEC tube formation, proliferation and migration post-miR overexpression *in-vitro*. Increased vascular defects in miR-24–overexpressing zebrafish compared with controls. Increased capillary and arteriolar density post-miR-24 inhibition in a MI mouse model	PAK4 GATA2	Fiedler et al., 2011
miR-23-24-27 cluster *(miR-24-3p)*	HUVECs Male CD1 mice-HLI model induction by left femoral artery ligation	Decreased EC survival, tube formation and proliferation post-transfection with pre-miR-24-3p *in-vitro*. Opposite results post-miR inhibition *in-vitro*. Increased capillary density post-miR inhibition *in-vivo*, but decreased blood perfusion since the new vessels appeared disorganised and twisted	Notch1 Dll1	Marchetti et al., 2020
miR-26a	HUVECs flk:eGFP zebrafish embryos Male C57BL/6 mice- MI induction by LAD coronary artery ligation	Decreased HUVEC tube formation and migration post-miR overexpression *in-vitro*. Overexpression of miR-26a in zebrafish impaired the development of caudal vein plexus (CVP) axial vein. Reduced infarct size and increased number of CD31^+^ cells after miR-26a inhibition in mice post-MI	SMAD Family Member 1 (SMAD1)	Icli et al., 2013
miR-34	Male C57BL/6 mice- MI induction by LAD coronary artery ligation	Improved cardiac function, capillary density and left ventricle remodelling post-inhibition of miR-34 *in-vivo* compared with controls	Unknown	Bernardo et al., 2012
miR-17-92 Cluster *(miR-92a*)	Pig-ischaemia/reperfusion induction (percutaneous transluminal coronary angioplasty balloon was placed in the LAD artery distal to the first diagonal branch for 49-60 minutes MiR-92a–deficient mice- MI induction by LAD coronary artery ligation C57Bl/6 mice-MI induction by LAD coronary artery ligation & HLI induction by ligation of the superficial and deep femoral artery and vein	Increased capillary density and reduced cardiac inflammation and post-mir-92α inhibition in mouse and pig MI models and in a HLI mouse model compared with controls	INTGA5	Bonauer et al., 2009; Hinkel et al., 2013; Bellera et al., 2014
miR-100	HUVECs C57/Bl6J mice- HLI induced by double ligation of the superficial femoral artery proximal to the deep femoral artery and distal femoral artery	Increased HUVEC tube formation, proliferation and migration post-miR inhibition *in-vitro*. Opposite effects were observed post-miR-overexpression *in-vitro*. Increased perfusion and capillary and arterial density post-miR-100 inhibition *in-vivo* compared with controls	Unknown	Grundmann et al., 2011
miR-124	HUVEC Male C57BL/6 mice- thoracic aorta constriction (TAC)	Increased HUVEC tube formation, proliferation and migration post-miR inhibition *in-vitro*. Opposite effects were observed post-miR-overexpression *in-vitro*. Impaired cardiac function and blood flow in intravenous adeno-associated virus (AAV)-miR-124-injected mice compared with controls	CD151	Zhao et al., 2018
miR-132/212	HUVEC Mice- HLI induced by double ligation of the superficial femoral artery proximal to the deep femoral artery and distal femoral artery	Decreased tube formation and migration of EC *in-vitro* post-miRNA overexpression. Opposite effects were observed post-miRNA inhibition. Increased capillary and arterial density post-miRNA inhibition *in-vivo*	RAS p21 protein activator 1 (Rasa1) (previously identified target of miR-132) SPRED1 (target of both miRNAs) Spry1 (target of both miRNAs)	Lei et al., 2015
miR-135-3p	HUVEC	Increased HUVEC tube formation, proliferation and migration post-miR inhibition *in-vitro*. Opposite effects were observed post-miR-overexpression *in-vitro*	Huntingtin-interacting protein 1 (HIP1)	Icli et al., 2019b
miR-142a-3p	Tg(fli1:EGFP)y1 zebrafish embryos expressing GFP in ECs	Loss of vascular integrity, haemorrhage and vascular remodelling post-injection of miR-142a-3p in zebrafish embryos. Normal primary vasculature but defective intersegmental vessels (Se) and abnormal remodelling in miR-142a-3p knock-out embryos	Cdh5 (VE-cdh)	Lalwani et al., 2012
miR-143/145 cluster *(miR-143, miR-145)*	HUVECs	Increased HUVEC tube formation and proliferation post-miR inhibition. Opposite results post-miR-143/-145 overexpression *in-vitro*	Hexokinase II (HKII) (miR-143 target) Integrin β (miR-145 target)	Climent et al., 2015
miR-183 cluster *(miR-96, miR-182, miR-183)*	Mouse cardiac endothelial cells (MCECs) HCMECs C57BL/6N mice -MI induction by LAD coronary artery ligation miR-96/miR-183 knock-out mice -MI induction by LAD coronary artery ligation	Overexpression of miR-96 and/or miR183 reduced tube formation and proliferation, but no migration of neonatal MCECs *in-vitro*. Inhibition of miR-183 cluster improved tube formation, proliferation and migration of adult MCECs *in-vitro*. Inhibition of miR-96 and miR-183 increased tube formation and proliferation of adult MCEC *in-vitro*, whereas miR-182 inhibition did not affect these measures. Similarly, overexpression of miR-96 and miR-183 in HCMECs reduced tube formation and proliferation, whereas dual inhibition of these miRs increased both parameters. Injection of miR-96 and miR-183 mimics in neonatal mice post-MI decreased vascularisation around the fibrotic tissue and increased the retention of scar tissue. MI induction in miR-96/miR-183 knockout mice resulted in increased capillary and arteriole densities, nut cardiac function and fibrosis did not change significantly compared to the WT controls.	Anillin (ANLN)	Castellan et al., 2020
miR-185	HMVECs	Decreased HMVEC tube formation, proliferation and migration post-miR overexpression. Opposite results post-miR-inhibition *in-vitro*	Stromal interaction molecule 1 (STIM1)	Hou et al., 2016
miR-199a-5p	Bovine aortic endothelial cell (BAEC)	Increased EC tube formation post-miRNA inhibition *in-vitro*	VEGFA (target in HEK293 cells) Calcineurin (target in HEK293 cells) SOD1 (target in HEK293 cells	Joris et al., 2018
miR-214	EPCs HUVECs Mice-HLI induction by right femoral artery and the distal portion of saphenous artery ligation C57BL/6 mice- TAC restriction	Increased EPC tube formation post-miR inhibition. Increased HUVEC tube formation, proliferation and migration post-miR inhibition. Opposite results post-miR-214 overexpression *in-vitro*. Increased blood flow by transplantation of EPC, in which miR-214 was inhibited, to the ischaemic limb tissue. Improved cardiac function and increased number of capillaries post-AAV9-anti-miR-214 injection compared with controls	X-box binding protein 1 (XBP1)	Duan et al., 2015; Jin et al., 2015
miR-217	HUVECs HAECs HCAECs	Decreased EC tube formation and migration post-miR overexpression *in-vitro*. Opposite results post-miR-inhibition *in-vitro*	Silent Information Regulator 1 (SirT1) (target of miR-217)	Menghini et al., 2009
miR-221/222	HUVECs	Decreased HUVEC tube formation and migration post-miR overexpression *in-vitro*	Signal transducer and activator of transcription 5A (STAT5A) (miR-222 target)	Poliseno et al., 2006; Dentelli et al., 2010
miR-342-5p	HUVEC mouse aortas from endothelial-specific Notch-activating mice	Decreased HUVEC tube formation and migration post-miR overexpression *in-vitro*. Decreased EC sprouting in aortic rings post-miR mimic transfection ex*-vitro*	Endoglin	Yan et al., 2016
miR-483-5p	HUVECs	Decreased HUVEC tube formation and migration post-miR overexpression. Opposite results post-miR-inhibition *in-vitro*	Serum response factor (SRF)	Qiao et al., 2011
miR-615-5p	HUVEC C57BL/6 mice-HLI induced by femoral artery ligation	Increased HUVEC tube formation, proliferation and migration post-miR inhibition *in-vitro*. Opposite effects were observed post-miR-overexpression *in-vitro*. Improved blood flow recovery and capillary density post-miRNA inhibition *in-vivo*	IGF-2 Ras-associating domain family member 2 (RASSF2)	Icli et al., 2019a
miR-665	HUVECs Human cardiac microvascular endothelial cells (HCMVECs) Male C57BL/6 mice- TAC restriction	Decreased HUVEC tube formation, proliferation and migration post-miR overexpression *in-vitro*. Opposite results post-miR-inhibition *in-vitro*. Increased coronary microvessel density and improved heart function post-miR-665 inhibition *in vivo*	Ago2	Fan et al., 2018
miR-939	HUVECs	Decreased HUVEC tube formation, proliferation and adhesion, but increased migration post-miR overexpression *in-vitro*. Opposite results post-miR-inhibition *in-vitro*	γ-catenin	Hou et al., 2017
**miRNAs WITH OPPOSITE** ***IN-VITRO*** **AND** ***IN-VIVO*** **FUNCTIONS**				
miR-146a	HUVEC Balb/c mice-femoral artery ligation	Impaired EC tube formation and proliferation post-miRNA inhibition *in-vitro. In vivo*, miRNA inhibition post-femoral artery ligation did not affect capillary density but significantly increased collateral artery diameter (arteriogenesis)	Unknown	Heuslein et al., 2018
miR-155	HUVECs Aortic rings from miR-155^−/−^ mice C57/BL6J mice- HLI induction by double ligation of the superficial femoral artery proximal and distal to the deep femoral artery miR-155^−/−^ mice- HLI induction by double ligation of the superficial femoral artery proximal and distal to the deep femoral artery	Increased HUVEC tube formation and proliferation post-miRNA inhibition *in-vitro*. Decreased EC sprouting in aortic rings from miR-155^−/−^ mice. Decreased blood flow recovery post-ischaemia in miR-155-deficient mice compared to controls	Angiotensin II receptor type I (AGTR1)	Pankratz et al., 2015

On the other hand, several miRNAs have an inhibitory role in post-ischaemic neovascularisation. Overexpression of another component of cluster miR-23-24-27, miR-24, significantly impaired angiogenesis in zebrafish embryos by targeting p21 activated kinase (PAK4) and global transcription factor binding protein 2 (GATA2). Inhibition of this miRNA with chemically engineered cholesterol-conjugated single-strand RNA analogues (antagomirs) in a MI mice model increased capillary and arteriolar density, and improved cardiac function 14 days post-MI (Fiedler et al., 2011). The miRNA 17-92 cluster also has a prominent role in the regulation of post-ischaemic neovascularisation. Overexpression of the miR-92a component of this cluster has been reported to block angiogenesis *in-vitro* and *in-vivo*. Among the verified targets of miR-92a, integrin alpha 5 (ITGA5) critically influences EC proliferation and migration (Bonauer et al., 2009; Doebele et al., 2010; Daniel et al., 2014). Interestingly, antimir-92a (MRG-110) was evaluated in clinical trials; the results showed increased angiogenesis and blood perfusion following intradermal injection at the site of a small skin wound in healthy volunteers, as well as reduced alpha-smooth muscle actin (α-SMA) expression, which is correlated with activation of myofibroblasts (Safety Tolerability Pharmacokinetics, 2018).

## EVs in Neovascularisation

Over the past few years, EVs have emerged as novel regulators of therapeutic processes, including cardiac neovascularisation due to their ability to transfer molecules, such as miRNAs, in ECs. EVs are defined as heterogeneous plasma membrane vesicles and are released in the extracellular space under normal and pathological conditions (Raposo and Stahl, 2019). According to their size and biogenesis pathway, they can be classified into three main types: exosomes, microvesicles and apoptotic bodies.

Exosomes are a class of cell-derived EVs of endosomal origin that are typically 30–150 nm in diameter and contain various macromolecules derived from the cell of origin. These include miRNAs, proteins, lipids and mRNAs (Isola and Chen, 2016). Exosome biogenesis starts from the intraluminal budding of endosomal compartments. This forms intraluminal vesicles (ILVs) in the endosomal compartments, which are known as multivesicular bodies (MVBs). MVBs can either fuse with lysosomes for subsequent degradation or fuse with the plasma membrane releasing the ILVs, in the extracellular space as exosomes. In contrast to exosomes, microvesicles, are typically 100–1,000 nm in size and are formed by the outward blebbing of the plasma membrane. During the blebbing process, disruption of the actin cytoskeleton and membrane reorganisation occurs (Cocucci and Meldolesi, 2015). The modification of membrane asymmetry results in the redistribution of aminophosholipids to the outer part of the cell membrane. Interestingly, microvesicle formation seems to occur in lipid-rich microdomains of the plasma membrane, such as lipid-rafts or caveolae domains (Del Conde et al., 2005; Morel et al., 2009). On the other hand, apoptotic bodies are exclusively formed during the last steps of apoptosis and range from 800 nm to 5 μm (Caruso and Poon, 2018). Investigating the mechanisms of EV release and uptake by ECs is critical to understand their role in neovascularisation under pathological conditions, such as MI. Recently, a new population of non-membranous nanoparticles ~35 nm in size, termed “exomeres” was identified (Zhang H. et al., 2018; Zhang et al., 2019). Zhang H. et al. (2018), recently showed that exomeres have a distinct protein, lipid, DNA and RNA profile to exosomes, and demonstrate unique organ distribution patterns, suggesting different biological functions. In contrast to EVs, exomere biogenesis remains unclear.

### EV Release by ECs

EV release is a complex process that involves cytoskeletal proteins (actin and microtubules), molecular motors (kinesins and myosins), as well as molecular switches (small GTPases) and the fusion machinery (SNAREs and tethering factors) (Raposo and Stoorvogel, 2013). Ras-related proteins in brain (RAB) family, including Rab11, Rab27a/27b, and Rab35 coordinate membrane trafficking events, and have emerged as essential components of exosomal release in several cell types (Savina et al., 2002; Hsu et al., 2010; Ostrowski et al., 2010). Rab7 is another member of RAB family that mediates the maturation of late endosome and mediates their fusion with lysosomes (Vanlandingham and Ceresa, 2009). Jaé et al. (2015), showed that Rab7 and Rab27b regulate the secretion of endothelial miRNA through EVs. Moreover, Ostrowski et al. (2010), demonstrated that knocking down the Rab27a/27b effectors synaptotagmin-like 4 (SYTL4) (also known as Slp4) and exophilin 5 (EXPH5) also inhibits exosome secretion in HEK293 cells. Interestingly, Slp4 was also shown to have a central role in Weibel-Palade body (WPB) exocytosis from ECs (Bierings et al., 2012).

Although our understanding of the exact mechanism of EV-release from EC is lacking, several factors have been shown to trigger the release of EVs from ECs. EC injury is a critical part of the development of CHD and significantly affects the levels of EC-derived EVs (Werner et al., 2006). In general, EC-EVs are present at lower concentrations under physiological conditions and, upon activation, are released from ECs (Koga et al., 2005). Elevated levels of plasma EC microparticles have been reported in several CVD, including CHD (Koga et al., 2005; Werner et al., 2006; Nozaki et al., 2009). Moreover, clinical studies in heart failure patients have revealed that the number of circulating EVs from ECs and EPCs greatly depends on the severity of heart failure. Berezin et al. (2016), for instance, showed that heart failure patients with preserved ejection fraction had an increased number of apoptotic EC-derived EVs and less activated EC-derived EVs than patients with heart failure with reduced injection fraction. Interestingly, EC-derived EVs have been proposed as novel biomarkers of EC dysfunction and may determine the risk of acute heart failure (Horstman, 2004).

EVs of endothelial origin have been found to play a versatile role in neovascularisation since their effect seems to be affected by the dose used. Lacroix et al. (2007), for instance, showed that low amounts of microparticles (2 × 10^3^ particles/well, in a 96-well plate) from TNFa-stimulated HMVECs could increase tube formation of endothelial progenitor derived cells (EPDCs) *in-vitro*. In contrast, high amounts of microparticles (2 × 10^5^ particles/well, in a 96-well plate) had an inhibitory effect. Liang et al. (2018), showed that hypoxic HUVEC EVs could also inhibit EC migration and angiogenesis probably due to increased levels of miR-19b in these EVs (5 x 10^4^ EVs/well, in a 6-well plate were used). The ability of ECs to stimulate angiogenesis may involve the transfer of miR-214 (Balkom et al., 2013). Interestingly, Chen et al. (2018), showed that EPC-derived EVs could increase angiogenesis but not proliferation *in-vitro* (7 × 10^9^ EVs, in a 24-well plate). The intramyocardial delivery of EPC-derived EVs incorporated into shear-thinning hydrogels in Wistar rats, could increase angiogenesis, preserve ventricular geometry and improve haemodynamic function post-MI (9.33 × 10^9^ EVs were delivered via 5 × 20 μl injections around the border zone of the infarcted area). Ou et al. (2011), however, demonstrated that endothelial microparticles from HUVECs in increased concentrations (higher than 10^5^ microparticles/ml) could inhibit angiogenesis in mouse heart sections. These data suggest that EC release EVs with diverse roles in neovascularisation.

### EV Uptake by ECs

EVs transfer information to the recipient cells by various mechanisms, including clathrin-mediated endocytosis, micropinocytosis, phagocytosis, caveolin-mediated endocytosis, and lipid raft mediated endocytosis (Mulcahy et al., 2014). The glycoproteins [e.g., Heparan sulphate proteoglycans (HSPG) (Christianson et al., 2013)] and proteins [e.g., integrins (Morelli et al., 2004), tetraspanins (Hemler, 2005)] on the surfaces of EVs and their target cells are recognised as critical factors that determine the uptake mechanism. Nazarenko et al. (2010), showed that treating exosomes with antibodies against tetraspanin-8, integrin CD49d, and vascular cell adhesion molecule-1 (VCAM-1, CD106) significantly reduced exosome uptake by RAECs. However, the precise mechanisms of EV uptake by ECs is still unknown. Some EVs can deliver their content to ECs through clathrin-mediated endocytosis. Dynamin is a GTPase essential for membrane fusion during this process. Blocking dynamin activity is a common strategy to study this mechanism (Singh et al., 2017). Chiba et al. (2018), showed that pancreatic cancer cell exosomes may be transferred to ECs through dynamin-dependent clathrin-mediated endocytosis, resulting in increased angiogenesis *in-vitro*. This was verified by blocking dynamin activity with a small inhibitor, called dynasore. Moreover, Svensson et al. (2013), reported that internalisation of exosomes derived from glioblastoma cells by ECs is significantly decreased post-methyl-β-cyclodextrin (MβCD) treatment. MβCD is a water-soluble oligosaccharide able to remove cholesterol from cell membranes, suggesting that lipid-raft endocytosis is a critical mechanism of EV uptake.

The most commonly used method for detecting EV uptake by ECs *in-vitro* and *in-vivo* is by using fluorescent lipid membrane dyes that stain EV membranes, such as PKH67 (Balkom et al., 2013), PKH26 (Lopatina et al., 2014; Lombardo et al., 2016; Zou et al., 2016; Mao et al., 2019) and DiI (Liang et al., 2016). Lopatina et al. (2014), for instance, showed that PKH26-stained MSC-EVs could be internalised into HMVECs and promote angiogenesis *in-vitro* and *in-vivo*. Moreover, Liang et al., demonstrated that the uptake of DiI-labelled MSC-exosomes by HUVECs increased tube formation *in-vitro*. To assess the role of MSC exosomes in EC angiogenesis *in-vivo*, the authors performed Matrigel plug assays in mice subcutaneously injecting HUVECs mixed with MSC-exosomes and HUVEC alone, and consistently with their *in-vitro* data, they showed that MSC-exosomes resulted in increased angiogenesis *in-vivo*. One potential issue with lipid-bound dyes is the leaching of the fluorescent molecules onto the cellular membranes, which can lead to a pattern of internalisation due to membrane recycling rather than EV uptake. To distinguish between surface-bound and internalised fluorescent EVs, the surface of the cell can be treated with trypsin (Franzen et al., 2014). A further limitation of these dyes is that the presence of the fluorescent molecules may affect the physical properties and thus, the normal behaviour of EVs. Membrane permeable dyes, such as carboxyfluorescein succinimidyl ester (CFSE) and 5(6)- carboxyfluorescein diacetate (CFDA), that are confined to the cytosol and fluoresce as a consequence of esterification, as well as Calcein AM are also used to study EV uptake by ECs (Teng et al., 2015; Li et al., 2018; Merckx et al., 2020). Radionuclides and magnetic particles have also been exploited to label EVs. Lee et al. (2020), for instance, recently showed that nanovesicles derived from iron oxide NP-incorporated MSCs could be effectively targeted to the myocardium of rats promoting cardiac function and angiogenesis. Finally, by fusing Gluc to a protein enriched in the membrane of exosomes, such as lactadherin, exosomes can emanate a strong luminescent signal when a Gluc substrate is present (Zhang K. et al., 2018). EVs can also be labelled with GFP by expressing a DNA construct coding for EV markers such as CD63, CD81, and CD9 fused to GFP in parent cells. This way, EVs can be tracked or purified to study their cargo (Garcia et al., 2015; Ribeiro-Rodrigues et al., 2017). Nonetheless, visualising GFP-labelled EV uptake by conventional fluorescent microscopy techniques is challenging due to the small nature of EVs. For this reason, the use of advanced technologies, including electron microscopy and atomic force microscopy are useful in EV characterisation (Mondal et al., 2019).

Recently, Cre-loxP system was also introduced as a very promising strategy to study EV uptake by cancer cells (Zomer et al., 2016). Adapting this system, de la Cuesta et al. (2019), visualised direct transfer of human pulmonary artery smooth muscle cells (HPASMCs) EVs to human pulmonary arterial endothelial cells (HPAECs). In particular, donor HPASMCs were transduced with a Cre recombinase lentiviral vector and HPAECs with a reporter lentiviral vector, that carried DsRed and eGFP separated by a loxP site. Cells were co-cultured in Boyden chambers, being physically separated by a membrane to prevent direct cell-cell contact. Zomer et al. (2015), previously showed that in this system, only *Cre* mRNA and not Cre protein is transferred into EVs. By performing qRT-PCR de la Cuesta et al. (2019) confirmed efficient loading of *Cre* mRNA into HPASMC-EVs. Using this system, the authors showed that HPASMC-EV-mediated Cre recombination resulted in a colour switch from red to green in reporter^+^ HPAECs, effectively evidencing communication via EVs. Therefore, the advantage of this approach is that EV uptake can be visualised in a more sensitive manner since it results in the fluorescent labelling of whole cells instead of small EVs.

### Animal Models to Study EV Trafficking

One of the greatest limitations in using purified labelled EVs from cell culture supernatants into the circulation of animal models is the difficulty to translate the *in-vivo* implications of EVs at a functional level. The variations observed in studies using EVs from different cell sources, the number of particles injected, the route of administration as well as the fact that most studies are implemented in immunodeficient animals, are all important variables in the design and analysis of experimental data. For this reason, genetically engineered mouse models (GEMM) could provide a promising approach to outreach current limitations allowing tracing of EVs in living organisms. As previously mentioned, a new strategy that allows the study of EV transfer *in-vitro* and *in-vivo* using the Cre/loxP system was reported (Zomer et al., 2016). This approach involves the fluorescent labelling of Cre-reporter cells that take up the EVs released from cells which express Cre recombinase. In this method, prior to cell injection into mice ubiquitously expressing the Cre-LoxP reporter tdTomato (tdTomato B6 mice), cells were transfected with a plasmid carrying Cre recombinase and cyan fluorescence protein (CFP). In this way, donor cells expressing Cre recombinase were CFP positive (blue), and EVs derived from these cells carried *Cre* recombinase mRNA. Upon injection, EVs were taken up by recipient reporter cells that translated *Cre* mRNA into protein. This resulted in the recombination of the reporter gene and activation of GFP expression leading to a colour switch from red to green fluorescence exclusively in cells that had taken up functional EVs *in-vivo* Zomer et al. (2015), developed this system to study EV communication between tumour cells. EV-miRNA profiling revealed that malignant tumour cells contain mRNAs involved in tumour migration and metastasis. More importantly, intravital imaging, showed that EVs released by malignant cells can be internalised by less malignant cells within the same or distal tumours and that the less malignant tumour cells display enhanced migratory behaviour and metastatic capacity. The exact mechanism of how the *Cre* mRNA is transferred into exosomes remains unclear, but it is probably a simple reflection of increased cellular expression and sufficient EV loading. Interestingly, the authors verified that the colour switch was due to *Cre* mRNA containing EVs, and not by other mechanisms, such as free *Cre* mRNA or protein.

Other strategies involve the controlled expression of *Cre* recombinase under specific promotors. For example, *Cre* mRNA can be expressed selectively in haematopoietic cells under the vav1 promoter and sorted into EVs released into the bloodstream. Upon entering a target cell, this mRNA is translated to a functional protein, leading to excision of the stop-loxP site and induction of marker gene expression in transgenic mice. Based on this, Kur et al. (2020), proved that neuronal activity triggers the uptake of haematopoietic extracellular vesicles *in vivo*. In particular, they showed that after the induction of peripheral inflammation by intraperitoneal (IP) injection of lipopolysaccharide (LPS), there were frequent recombination events in the hippocampus, substantia nigra, and other regions of the brain as observed by yellow fluorescent protein (EYFP) expression. EYFP expression was mediated by Cre recombinase activity, indicating that haematopoietic cell EVs were transferred and internalised in these regions of the brain. Although most studies utilising GEMM to assess EV uptake are focused on cancer or neuroscience research, the adaptation of these animal models could greatly increase our knowledge in EV communication in cardiovascular disease, including MI. The use of the Cre-loxP system has significantly increased our understanding of EV communication. However, the fact that *Cre* mRNA can be loaded in and transferred through EVs may doubt the validity of approaches using the Cre-loxP system in a tissue-specific manner, since upon activation of *Cre* expression in specific cells, *Cre* mRNA or protein can be transferred to other cell types through EVs, leading to unfavourable recombination events.

Recently, McCann et al. (2020), generated a reporter mouse bearing a CD63-emGFP^loxP/stop/loxP^ knock-in cassette that enables the labelling of cell type-specific EVs *in-vivo*, without prior *in vitro* manipulation. Upon crossing with a lineage-specific Cre recombinase driver mice, this system enables the specific labelling of circulating CD63^+^ vesicles from the cell type of interest. By crossing the mice bearing the CD63-emGFP^loxP/stop/loxP^ knock-in cassette with Cdh5-Cre^ERT2^ mice, the authors generated CD63emGFP+ vasculature and showed that following tamoxifen administration to pregnant females the developing vasculature of the embryos was marked with emerald GFP (emGFP). Most importantly, whole plasma-purified EVs contained a subpopulation of emGFP^+^ vesicles that co-expressed EV markers, including CD9 and CD81, and EC markers, like CD105.

Altogether, the recent development of genetically modified mouse models to study EV trafficking *in-vivo* holds great promise as valuable tools for unravelling the *in-vivo* relevance of EVs in physiological and pathophysiological processes such as cardiac neovascularisation after cardiac ischemia.

### EVs in Therapeutic Neovascularisation

EVs possess inherent tissue repair properties that make them ideal candidates for regenerative medicine therapeutics. Several studies have demonstrated the ability of EVs to promote neovascularisation post-ischaemia. Gallet et al. (2017), showed that intramyocardial delivery of cardiosphere-derived cell (CDC) exosomes in a pig MI model increased vessel density and cardiomyocyte hypertrophy, while preserving left ventricular (LV) and left ventricular ejection fraction (LVEF) volumes and reducing scar size. Moreover, Potz et al. (2018), showed that intramyocardial injection of human mesenchymal cell-derived EVs in a swine MI model could increase blood flow, capillary and arteriolar density 5 weeks post-left circumflex artery ligation. Chen et al. (2020), aimed to investigate the role of exosomes derived from remote ischemic conditioning (RIC) in cardiac remodelling and function. Exosomes were isolated from the plasma of rats subjected to HLI and injected to the caudal vein of rats once every 3 days post-MI. The authors showed that RIC and RIC exosomes significantly improved cardiac function and blood vessel formation, and decreased collagen deposition 28 days post-MI. Wu et al. (2020), demonstrated that the intramyocardial injection of EVs from ESC-derived cardiovascular progenitor cells (CVPCs) cultured under normoxia or hypoxia could significantly improve cardiac function, vascularisation and cardiomyocyte survival, and reduce fibrosis at 28 days in a mouse MI model. More importantly, EVs secreted from ESC-CVPC cultured under hypoxia had a better benefit in improving cardiac function post-MI.

EVs possess several advantages over cell-based therapeutics and conventional delivery systems. A major advantage is that EVs may be less immunogenic than their parental cells, probably due to the presence of less membrane-bound proteins like MHC complexes on their surface (Ong and Wu, 2015). The number of MHC molecules on EV surface highly depends on the cell of origin and the EV subtype (Wahlund et al., 2017). Recently, Kompa et al. (2020), used a subcutaneously implanted TheraCyte device for sustained delivery of the secretome of human cardiac stem cells (hCSCs) in a rat MI model. Cells can be enclosed in the TheraCyte device, being protected by the host's immune system while allowing the therapeutic secreted products to freely diffuse from within the device. The authors showed that hCSC secretome could preserve LV ejection fraction and cardiac function, reduce fibrotic scar tissue, interstitial fibrosis and cardiomyocyte hypertrophy, while increasing vascular density. To visualise the EV transfer from hCSC to the myocardium, they used CSCs expressing plasma membrane reporters and confirmed that EVs from W8B2^+^ CSCs could be transferred to the heart and other organs 4 weeks post-implantation. A further advantage of EVs is that they can be easily stored, retaining their function over prolonged periods, overcoming many limitations of the use of viable cells in regenerative medicine. Moreover, EVs are naturally occurring lipid nanoformulations that, compared to other synthetic drug delivery systems, may be promising carriers of therapeutic molecules, exhibiting less toxicity and increased stability under both physiological and pathological conditions. EVs might also be combined with other strategies to optimise therapeutic agent delivery. Liang et al. (2017), showed that exosomes could naturally envelope AAV-vectors (AAVExo) and protected from plasma neutralising antibodies, AAVExo could transduce cardiomyocytes with higher efficiency than free AAVs in a mouse MI model. For these reasons, EVs have emerged as ideal carriers for the delivery of therapeutic molecules, such as miRNAs, to ECs for the promotion of neovascularisation post ischaemia.

### EV-bound miRNAs in Neovascularisation

EVs exert their action through the transfer of molecules, such as small RNAs and proteins, able to control molecular pathways in the recipient cells once transferred. For this reason, they have gained immense interest as therapeutic vehicles. Among their multidimensional role, several studies have demonstrated the ability of EVs to stimulate vascular growth and maturation by delivering pro-angiogenic miRNA molecules to ECs. Table 2 contains a list of EV-bound miRNAs as reported in EV-based neovascularisation studies. However, since our intention was to identify EV-bound miRNAs with a role in neovascularisation, this table contains a simple summary of our findings with an emphasis on the miRNAs, and due to space constraints, not all EV-producing cell types are reported.

**Table 2:** EV-bound miRNAs in the regulation of cardiovascular neovascularisation.

**EV miRNA**	**Model**	**Effect**	**EV source**	**EV isolation method**	**References**
miR-15b miR-17 miR-20a miR-103 miR-199a miR-210 miR-292	Rat cardiac endothelial cells Male Sprague-Dawley rats- MI induction by LAD coronary artery ligation	Increased EC tube formation post-hypoxic EV treatment *in-vitro*. Improved cardiac function and reduced fibrosis post-intramyocardial EV injection at 3 border zones. EV microRNA expression profiling by qPCR miRNA array revealed several upregulated miRNAs under hypoxic conditions	Normoxic/ hypoxic CPCs	Differential centrifugation ultracentrifugation	Gray et al., 2015
miR-16 miR-17-92 miR-19b miR-20a miR-34 miR-126-3p miR-130a-3p miR-210-3p miR-294	Murine cardiac ECs. C57BL6/J mice- MI induction by 30-min coronary occlusion followed by reperfusion	Increased EC tube formation, migration and antiapoptotic properties post-EV treatment *in-vitro*. Increased capillary density & reduced LV remodelling and hypertrophy post-intramyocardial injection of EVs at 5 sites at the border between infarcted and non-infarcted myocardium 48h post-reperfusion. EV microRNA expression profiling was performed by miRNA array and revealed several upregulated miRNAs.	iPSCs	Differential centrifugation ultracentrifugation	Adamiak et al., 2018
miR-17 miR-19a miR-19b miR-20a miR-30c miR-126	HUVECs	Increased EC tube formation, proliferation and migration post-exosome treatment *in-vitro*. EV microRNA expression profiling was performed by qPCR miRNA array and revealed increased levels of several miRNAs in these exosomes	Glucose starved H9C2 cardiomyocytes	Differential centrifugation ultracentrifugation	Garcia et al., 2015
miR-20b miR-27b miR-29b miR-42a miR-100 miR-125b miR-143 miR-195 miR-291b miR-497	CMVECs Aortic rings from Male Sprague-Dawley rats Male Sprague-Dawley rats- HLI induction by femoral artery ligation	Increased EC tube formation, proliferation and migration post-exosome treatment *in-vitro*. Increased EC sprouting post-exosome treatment *ex-vivo*. Increased capillary density and blood perfusion post-induced vascular progenitor cell (iVPC) exosome injection as compared with rat aortic endothelial cell (RAEC) exosomes and controls. EV microRNA expression profiling was performed by qPCR miRNA array and revealed increased levels of several miRNAs in these exosomes	iVPCs RAECs	Ultracentrifugation Ultrafiltration Size-exclusion chromatography	Johnson et al., 2019
miR-21-5p	HUVECs Female CD-1 mice- MI induction by LAD coronary artery ligation	Improved EC tube formation *in-vitro* post-treatment with EVs from patients with normal angiography results (NEXO) compared to patients with heart failure (FEXO). Increased capillary density, infarcted wall thickness and decreased infarct size post-NEXO EV treatment compared to FEXO EVs and PBS controls post-intramyocardial injection. Decreased miR-21-5p expression in FEXO compared to NEXO group identified by EV microRNA expression profiling by qPCR miRNA array. Silencing of miR-21-5p in NEXO group decreased tube-formation *in-vitro*, while upregulation of miR-21-5p in FEXO group promoted EC tube formation.	Explant-derived cardiac stromal cells from FEXO or NEXO patients	Ultracentrifugation	Qiao et al., 2019
miR-21 miR-27a miR-29a miR-126 miR-130α miR-191 miR-210 miR-296-3p	HMVECs Human macrovascular endothelial cells (HMAVECs) SCID mice	Increased EC tube formation and proliferation post-treatment with EVs from obese individuals with type 2 diabetes (OD) compared with EVs from healthy individuals (H), patients with type 2 diabetes (D), obese individuals without T2DM (O), and patients with ischaemic disease (IC). EVs from patients of the above groups were divided as “effective” and “ineffective” depending on their effect on angiogenesis *in vitro*. Matrigel plug *in vivo* assays using EVs defined as “effective” in the *in vitro* assays, showed that these EVs resulted in an increased number of vessels in mice Matrigel plugs. EV microRNA expression profiling of “effective” and “ineffective” EVs of healthy individuals was performed by qPCR miRNA array and identified several angiogenic miRNAs	Human serum from H, D, OD, D, O, IC individuals	Ultracentrifugation	Cavallari et al., 2020
miR-31	HUVECs Aortic rings from male C57BL/6J mice	Increased EC tube formation and migration post-EV treatment *in-vitro*. Increased EC sprouting post-EV treatment *ex vivo*. EV microRNA expression profiling was performed by qPCR miRNA array and revealed increased levels of miR-31. EVs from cells transfected with anti-miR-31 decreased EC tube formation and migration *in-vitro*, whereas pre-miRNA transfection increased tube formation and migration.	Adipose-Derived Stem Cells (ADSCs)	Differential centrifugation Ultracentrifugation	Kang et al., 2016
miR-92a-3p	HCAEC	EV microRNA expression profiling was performed by qPCR miRNA array and revealed increased miR-92a-3p levels in oxidised low-density lipoprotein (oxLDL) and interleukin-6 (IL-6) stimulated HCAEC. Knock-down of this miRNA in HCAEC EVs decreased their ability to cause HCAEC migration, proliferation, and tube formation *in-vitro*.	oxLDL and IL-6 stimulated HCAEC	Differential centrifugation Ultracentrifugation	Liu et al., 2019
miR-125a-5p	HUVEC	Increased EC tube formation post-EV treatment *in-vitro*. EV microRNA expression profiling was performed by qPCR miRNA array. Overexpression of miR-125a in HUVECs, increased tube formation *in-vitro*. Matrigel plug *in vivo* assay results showed that the number of vascular structures was significantly higher in mice subcutaneously injected with HUVECs mixed with EVs than HUVECs alone	MSCs	Differential centrifugation Ultrafiltration Ultracentrifugation	Liang et al., 2016
miR-126-3p	HUVECs Immunocompromised BalbC mice- HLI induction by femoral artery ligation	Increased EC tube formation post-EV treatment *in-vitro*. EV microRNA expression profiling was performed by qPCR miRNA array. EVs from cells transfected with anti-miR-126-3p decreased HUVEC tube formation *in-vitro*. Improved blood flow and capillary density post-intramuscular injections of EVs at 4 different sites of the ischaemic limb.	CD34^+^ SCs	Ultracentrifugation Density gradient centrifugation	Mathiyalagan et al., 2017
miR-126 miR-296	SCID mice- HLI induction by left femoral artery and the distal portion of saphenous artery ligation	Increased capillary density and blood perfusion post-MV injection. Presence of miR-296/-126 in the EVs was confirmed by qPCR	EPCs	Ultracentrifugation	Ranghino et al., 2012
miR-132 miR-146a-3p miR-181 miR-210	HUVECs Male Wistar rats- MI induction by LAD coronary artery ligation	Increased EC tube formation ability post-EV treatment *in-vitro*. EV microRNA expression profiling was performed by qPCR miRNA array. HUVECs transfected with a miR-132 mimic had increased tube formation. Opposite results were observed post-transfection with si-miR-132. *In-vivo* experiments revealed increased blood vessel density post-intramyocardial injection of CPC EV at 3 border zones.	hCPCs	ExoQuick™ precipitation solution (System Biosciences; SBI) Ultracentrifugation column precipitation using Exo-spin™ kits (Cell Guidance Systems, Cambridge, UK)	Barile et al., 2014
miR-143 miR-222	MCECs HUVECs Rat aortic rings Fertilised chicken eggs Female C57BL/6 mice- MI induction by LAD coronary artery ligation	EVs from cells in ischaemic solutions increased EC tube formation, proliferation and protection against oxidative-induced lesion *in-vitro* and increased EC sprouting *ex-vivo*. Intramyocardial injection of hypoxic EVs increased capillary density, the number of CD31+ cells in the infarcted region and blood perfusion. EV microRNA expression profiling by qPCR miRNA array revealed increased miR-222/-143 expression in hypoxic EVs	H9c2 myocardial cells & primary rat cardiomyocytes from Wistar rat foetus cultured in appropriate media or ischaemia-mimetic solutions	Differential centrifugation Density gradient centrifugation	Ribeiro-Rodrigues et al., 2017
miR-210	HUVEC Male C57BL/6 mice- MI induction by LAD coronary artery ligation	Increased EC tube formation & decreased apoptosis post-hypoxic EV treatment *in-vitro*. EV microRNA expression profiling by qPCR miRNA array revealed increased miR-210 expression in hypoxic EVs. Transfection of HUVECs with miR-210 mimic yielded similar results to hypoxic EV treatment. Increased capillary and arteriole density and CM survival post-intramyocardial injection of hypoxic EVs at five sites around the border zone of infarcted hearts.	Normoxic/ hypoxic MSCs	Differential centrifugation Ultracentrifugation	Zhu et al., 2018
miR-214	HMVECs	Increased EC tube formation and migration post-EV treatment *in-vitro*. EV microRNA expression profiling was performed by qPCR miRNA array and showed increased miR-214 expression. EVs from cells transfected with anti-miR-214 decreased EC tube formation and migration *in-vitro*, whereas pre-miRNA transfection did not affect tube formation and migration.	HMVECs	Differential centrifugation Density gradient centrifugation	Balkom et al., 2013
miR-423-5p	HUVECs	Increased EC tube formation, proliferation and migration post-exosome treatment *in-vitro*. EV microRNA expression profiling by small RNA sequencing and qPCR miRNA array revealed increased levels of miR-423-5p in these exosomes. Overexpression of miR-423-5p in HUVECs increased their tube formation ability, proliferation and migration.	hADSCs	Differential centrifugation Ultracentrifugation	Xu et al., 2019
miR-939-5p	MCECs HUVECs C57BL/6 mice- MI by LAD coronary artery ligation & HLI by left femoral artery ligation	Increased EC tube formation, proliferation and migration post-ischaemic exosome treatment *in-vitro*. Increased capillary density and reduced scar size post-ischaemic exosome injection. EV microRNA expression profiling by qPCR miRNA array revealed decreased miR-939-5p expression in ischaemic exosomes. HUVEC transfection with miR-939-5p mimic decreased EC tube formation and migration *in-vitro*, whereas transfection with miRNA inhibitor increased tube formation and migration.	Coronary blood from patients with myocardial ischaemia and control group	Differential centrifugation Ultracentrifugation	Li et al., 2018
miR-1246	HUVECs	Incubation of HUVECs with DLD-1 cancer cell derived EVs significantly increased tube formation and migration. Mir-1246 was enriched in DLD-1-EVs. Overexpression of miR-1246 in HUVECs significantly increased their tube formation ability and migration.	DLD-1 cancer cells	Ultracentrifugation	Yamada et al., 2014
miR-4306	HCAECs	Decreased EC tube formation, migration and proliferation post-treatment with EVs isolated from the PCI group compared with those from the control group. Human monocyte-derived macrophage (HMDM) treatment with ox-LDL significantly increased their miR-4306 levels. Transfection of HCAECs with miR-4306 mimic significantly inhibited their tube formation ability and slightly suppressed HCAEC proliferation and migration.	HMDM from patients who had PCI performed within 12 hours (PCI group) and patients with chest pain syndrome with normal coronary artery findings post coronary angiography	Differential centrifugation Ultracentrifugation	Yang et al., 2019
let-7b-5p	HUVECs CD1 male mice-HLI induction by left femoral artery ligation	Increased EC tube formation, proliferation and reduced EC apoptosis post-exosome treatment *in-vitro*. EV microRNA expression profiling was performed by qPCR miRNA array and revealed increased levels of let-7b-5p. Exosomes restored the angiogenic capacity of DICER knock-out-ECs, but the reduction of exosomal let-7b-5p in exosomes failed to induce tube formation in recipient DICER-KD ECs *in-vitro*. Increased capillary density and blood flow recovery and reduced necrosis post-exosome injection *in-vivo*.	Human pericardial fluid (PF) exosomes	ExoQuick kit (System Biosciences)	Beltrami et al., 2017

**Figure 2:**
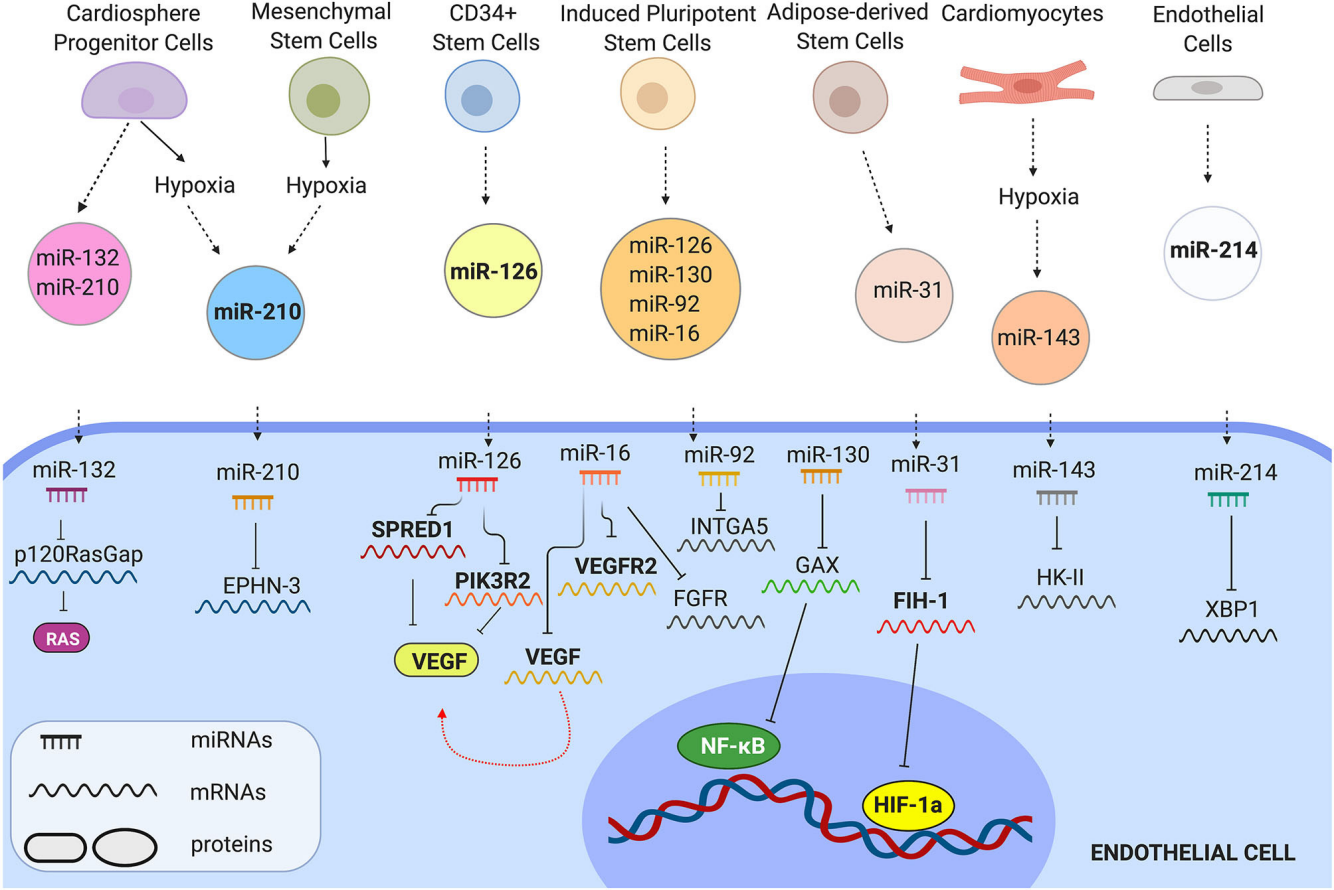
Representative examples of EV miRNAs that control EC behaviour. EVs from different cells carry angiogenic miRNAs. Once transferred in ECs, miRNAs control molecular pathways by inhibiting their mRNA-targets.

EV-bound miRNA molecules can be internalised in the cytoplasm of recipient cells and activate molecular pathways controlling cell behaviour (Figure 2). Barile et al. (2014), for instance, showed that EVs from human cardiac progenitor cells (hCPCs) were enriched in the angiogenic and cardioprotective miR-210, miR-132, and miR-146a-3p. As a result, EVs derived from these cells could significantly increase cardiac angiogenesis in a MI rat model. Wang et al. (2017), demonstrated that MSC-derived EVs were also enriched in miR-210. Treatment with MSC-EVs significantly increased HUVEC tube formation, proliferation and migration *in-vitro* and capillary density of matrigel plugs implanted in a mouse MI model by targeting Efna3. Silencing of miR-210 in MSC-EVs significantly impaired the *in-vitro* and *in-vivo* angiogenic effects. Adamiak et al. (2018), showed that treatment with iPSC-derived EVs, promoted murine cardiac EC tube formation, migration and antiapoptotic properties. Injection of iPSCs-EVs in a mouse MI model significantly increased capillary density & reduced LV remodelling and hypertrophy compared to the controls. EV microRNA expression profiling was performed by miRNA array and revealed that several miRNAs were upregulated in these EVs, including miR-16, miR-17-92, miR-19b, miR-20a, miR-34, miR-126-3p, miR-130a-3p, miR-210-3p, miR-294.

Specific conditions, such as hypoxia greatly affect gene expression and favour the production of miRNAs with angiogenic properties. As a result, several studies have confirmed that hypoxia leads to the release of angiogenic EVs. Ribeiro-Rodriquez et al. for instance, demonstrated that exosomes secreted by H9c2 myocardial cells and primary cardiomyocytes cultured under hypoxic conditions significantly increased the formation of new functional vessels post-MI in rats in comparison with exosomes cultured under normoxic conditions. An analysis of the miRNA profile of these exosomes revealed that the pro-angiogenic miR-222 and miR-143 were significantly increased in the hypoxic exosomes providing evidence that these miRNAs may contribute to post-ischemic neovascularisation (Ribeiro-Rodrigues et al., 2017). Similarly, Zhu et al. (2018), showed that exosomes derived from hypoxic MSCs augmented neovascularisation in a MI mouse model. Exosome RNA profiling revealed 145 genes that were upregulated in the hypoxic exosomes compared to the normoxic ones, with miR-210 being one of the most abundant miRNAs. To investigate the role of miR-210 in hypoxic-EV induced angiogenesis, the authors used a miR-210 inhibitor to block the activity of miR-210 in MSCs and revealed that exosomes derived from these cells failed to induce angiogenesis *in-vitro*. Moreover, overexpression of miR-210 in ECs resulted in improved tube formation similar to that achieved by hypoxic exosomes. Gray et al. (2015), reported that treatment with hypoxic EVs derived from CPCs significantly increased rat cardiac EC tube formation compared to treatment with normoxic EVs. Injection of hypoxic EVs in a mouse MI model significantly improved cardiac function and reduced fibrosis. EV microRNA expression profiling revealed several upregulated miRNAs under hypoxic conditions, including miR-15b, miR-17, miR-20a, miR-103, miR-199a, miR-210, miR-292.

Qiao et al. (2019), compared the effect of exosomes isolated from explant-derived cardiac stromal cells from patients with normal angiography results (NEXO) to exosomes from patients with heart failure (FEXO). They showed that intramyocardial injection of exosomes from the NEXO group in a mouse MI model significantly increased capillary density and decreased the infarct size, while injection of exosomes from the FEXO group exacerbated cardiac function and left ventricular remodelling. Exosomes from the FEXO group exhibited reduced ability to promote HUVEC tube formation *in-vitro*. EV microRNA expression profiling revealed decreased miR-21-5p expression in the FEXO compared to the NEXO group exosomes. As a result, silencing of miR-21-5p in NEXO group decreased HUVEC tube-formation, while upregulation of miR-21-5p in FEXO group restored HUVEC tube formation. Li et al. (2018) showed that EC treatment with exosomes from coronary blood of patients with myocardial ischaemia significantly increased EC tube formation, proliferation and migration *in-vitro*. Intramyocardial injection of exosomes in a mouse MI model significantly increased capillary density and reduced scar size. EV microRNA expression revealed decreased miR-939-5p expression in exosomes from coronary blood of patients with myocardial ischaemia compared to exosomes from healthy controls. Transfection of ECs with miR-939-5p mimic decreased EC tube formation and migration *in-vitro*, whereas transfection with miRNA inhibitor increased tube formation and migration.

Beltrami et al. (2017), showed that human pericardial fluid exosomes increased EC tube formation, proliferation and reduced EC apoptosis. EV microRNA expression profiling revealed that let-7b-5p was increased in these exosomes. Moreover, human pericardial fluid exosomes restored the angiogenic capacity of DICER knock-out-ECs *in-vitro*, but the reduction of exosomal let-7b-5p in exosomes failed to induce tube formation in the recipient DICER-KD ECs. Exosome injection in a mouse HLI model significantly increased capillary density and blood flow recovery and reduced necrosis. EVs from CD34+ stem cells also appear to be regulators of angiogenesis. In particular, Mathiyalagan et al. (2017), demonstrated that CD34^+^ cell-derived exosomes are enriched in pro-angiogenic miRNAs, such as miR-126-3p. As a result, injection of these exosomes in an HLI mouse model increased miR-126-3p levels without affecting the endogenous synthesis of this miRNA, implying a direct transfer of exosomal miR-126-3p to the ischaemic limb. Moreover, it was suggested that miR-126-3p enhanced angiogenesis by suppressing its known target, SPRED1. Ranghino et al. (2012), reported that the angiogenic miRNAs miR-126 and miR-296 were upregulated in EPC-derived EVs. Injection of EPC-derived EVs in a mouse HLI model significantly increased capillary density and blood perfusion to the ischaemic muscle. Recently, Johnson et al. (2019), showed that exosomes from induced vascular progenitor cell (iVPC) could significantly increase EC tube formation, proliferation and migration *in-vitro* and EC sprouting of mouse aortic rings. Exosome injection in a rat HLI model significantly increased capillary density and blood perfusion compared with rat aortic endothelial cell (RAEC) exosomes and controls. EV microRNA expression profiling revealed increased levels of several miRNAs in these exosomes, including miR-20b, miR−27b, miR-29b, miR-42a, miR-100, miR−125b, miR-143, miR-195, miR-291b, miR-497. Taken together, several studies have reported a role of EV-bound miRNAs in post-ischaemic neovascularisation. Nonetheless, a better understanding of their mechanism of action in the recipient cells is essential before clinical application.

## Challenges and future Perspectives

The delivery of EVs, carrying therapeutic miRNA molecules, to the heart remains promising for the regulation of therapeutic neovascularisation post-MI, but there are several challenges to the field. Our knowledge of endogenous cell communication via EVs, for instance, both under physiological and pathological conditions is limited. Therefore, *in-vivo* visualisation of EV spatial and temporal release from the vascular wall, as well as of EV uptake by vascular ECs is of utmost importance for a better understanding of the biological function of EVs. To develop EV-based therapeutics, a more in-depth evaluation of EV pharmacokinetics *in-vivo* is also crucial. A critical factor that affects EV pharmacokinetics is the route of administration. Gallet et al. (2017), showed that CDC-derived exosomes resulted in improved vessel density and cardiac function post intramyocardial injection, but were ineffective after intracoronary injection. Therefore, preclinical models that allow for the study of endogenous EVs, as well as the biodistribution of exogenously administrated EVs, must be developed for a better understanding of their role in cardiac neovascularisation.

Moreover, clinical trials with EV products will only be possible with the improvement of isolation and purification techniques. The development of highly reproducible methods of isolating GMP-quality EVs, however, remains a great challenge in the field. Due to the lack of standardised cell culture and isolation methods, numerous studies, use relatively impure populations of EVs, which may affect angiogenesis assays, leading to wrong assumptions for their functionality. Lipoproteins are the most common contaminants from serum-containing medium (Yuana et al., 2014; Sódar et al., 2016). In this reason, the choice between culture medium containing EV-depleted serum and culture medium without serum needs to be considered. Serum starvation, however, has been reported to cause significant stress to the cultured cells, which subsequently leads to altered EV secretion (Witwer et al., 2013). Although this issue can be avoided by using EV-depleted serum, there are still several limitations associated with serum EV depletion strategies. Rigorous EV depletion of FBS using long ultracentrifugation protocols, for instance, is time-consuming and cannot remove small serum RNAs which can be mis-annotated as human RNAs (Wei et al., 2016). More recently introduced ultrafiltration-based strategies can overcome these limitations and result in solutions of extremely low EV and small RNA content (Kornilov et al., 2018). Another major dilemma in the EV field is the lack of a consensus methodology for the isolation of pure and intact EVs. Differential centrifugation is the most commonly used method for EV purification. Further purification from co-pelleted protein complexes and lipoproteins can be achieved by a density gradient. Nonetheless, this approach is associated with increased EV aggregation and usually results in fusion or disruption of the isolated EVs. Moreover, the optimal parameters of ultracentrifugation highly dependent on the type of centrifuge rotor used. Thus, it is essential that alternative approaches be used. Interestingly, a combination of ultrafiltration with size-exclusion chromatography has been proposed as a novel EV isolation approach that yields particles of improved purity and quality (Nordin et al., 2015).

Using standardised cell culture and EV isolation methods is extremely important in angiogenesis studies. Depending on the conditions that EVs have been generated and isolated, as well as the EV subtype and the dose used, they may exhibit a powerful proangiogenic or inhibitory effect. Moreover, since cell culture and EV isolation procedures affect the EV cargo, a more in-depth characterisation of the way that therapeutic molecules, such as miRNAs are sorted and released by EVs is essential before clinical application. EV-driven miRNA transfer post-MI is a new promising strategy for the promotion of neovascularisation. Numerous miRNAs including miR-126, miR-92a, miR-210, miR-27b, and miR-24 have been recognised as key regulators of post-ischaemic neovascularisation, but only a limited number have been identified in EVs. KRAS-MEK signalling has been proposed as an important regulator of miRNA sorting in EVs (McKenzie et al., 2016). However, the exact molecular mechanisms that drive miRNA transfer into EVs are not fully understood. Recently, a CRISPR-Cas-9 based system for single-cell detection of EV-mediated functional transfer of RNA was put forward (de Jong et al., 2020). This approach, termed CRISPR Operated Stoplight System for Functional Intercellular RNA Exchange (CROSS-FIRE), is based on activation of a fluorescent protein in recipient reporter cells upon functional delivery of specific sgRNAs, expressed in EV-donor cells. In general, there is a consensus that small RNA composition of cells differs from that in EVs, and emerging evidence supports that the presence of certain motifs on miRNAs may facilitate their transfer into EVs (Villarroya-Beltri et al., 2013; Koppers-Lalic et al., 2014).

Whether miRNA-Ago2 or miRNA-RISC complexes are present in EVs is of great interest to the EV field. Bound to Ago2, EV-miRNAs could efficiently assemble into functional RISCs for downregulation of their mRNA-target in the recipient cells. Interestingly, the entire RISC was reported to be present in EVs secreted from cancer cells, leading to efficient and rapid silencing of mRNA-targets at the recipient cells (Melo et al., 2014). An argument against the presence of Ago2 in EVs is that serum, a typical cell culture additive, contains a significant amount of non-vesicular Ago2 which may contaminate crude EV pellets prepared by standard EV isolation techniques, including ultracentrifugation of the conditioned medium at 100,000 × *g* (Huang et al., 2013). Size exclusion chromatography or density gradient can be used to separate vesicular from non-vesicular components of cell culture media (Prieto-Fernández et al., 2019). Jeppesen et al. (2019) recently used high-resolution density gradients to separate a crude small EV pellet into non-vesicular and vesicular fractions. Western blot analysis revealed that argonauts, including Ago2, were present in both fractions in media from Gli36 glioblastoma cells, but only in the non-vesicular fraction of media from DKO1 or MDA-MB-231 cells. A possible explanation of these findings could be that both DKO1 and MDA-MB-231 cells carry KRAS mutations. In contrast to this, several pathogens appear to promote Ago2 trafficking in EVs (Bukong et al., 2014; Mantel et al., 2016). Therefore, the ability to detect Ago2 in EVs may be a result of multiple factors, including the cell of origin, experimental conditions and detection methods. Tracing miRNA transfer from the recipient to the donor cells via EVs would significantly increase our knowledge on the way that EV-bound miRNAs affect EC behaviour and therefore, promote neovascularisation post-cardiac ischaemia. Although, several approaches have been proposed (reviewed in Mateescu et al., 2017), the detection and tracing of specific miRNAs in EVs remain challenging due to several issues, including limited probe specificity, limited signal per EV and poor signal-to-background ratios.

Currently, interest has been shifted toward engineering EV surface proteins and cargo for improved targeting (e.g., by the inclusion of peptides) and functionality. In this way, synthetic EVs may be loaded with known angiogenic miRNAs and target ECs to promote neovascularisation. One of the most common strategies used includes the loading of EV-producing cells with exogenous miRNAs and the transfection with plasmids expressing peptides of interest. Thus, EVs derived from these cells may carry therapeutic miRNAs and express targeting peptides on their surface. Alternatively, exogenous cargo can be directly loaded into EVs by several methods, including electroporation, heat-shock or freeze-thaw procedures, detergent treatment or sonication (de Abreu et al., 2020). However, further investigation is needed to define the stoichiometry required for therapeutic effects observed post-EV-miRNA transfer.

Although the focus of this review is on miRNAs, to date, several types of non-coding RNAs have emerged as key regulators of angiogenesis. Long non-coding RNAs (lncRNAs) for instance, may also control EC proliferation (e.g., MALAT1, H19, GAS5) or angiogenesis (MEG3, MANTIS) (Kok and Baker, 2019; Simion et al., 2019). Increasing evidence suggests that the interactions of lncRNAs with miRNAs play a critical role in the regulation of angiogenesis (Zhao et al., 2020). Moreover, lncRNAs may be selectively packaged into EVs and transferred between cells, improving cardiac function and vascularisation and reducing fibrosis post-MI (Wu et al., 2020). Circular RNAs (circRNAs) have also emerged as novel regulators of EC function and angiogenesis (e.g., CANRIL, CZNF292, Circ_0010729,Circ_0003575, Circ_0054633, Circ_0000109) (Zhang and Huang, 2020). CircRNAs can also be transferred to ECs through EVs. In the context of post-ischaemic neovascularisation, Dou et al. (2020), showed that following femoral artery ligation in VSMC specific SIRT1 transgenic (SIRT1-Tg) mice, blood flow and capillary density is significantly decreased due to the delivery of exosomal cZFP609 from VSMCs to ECs. Interestingly, the authors reported that cZFP609 may inhibit angiogenesis via blockade of HIF-1a nuclear translocation and hypoxia-induced VEGF-A expression in ECs. Integrating knowledge of miRNAs with other non-coding RNAs and the way that they are transferred between cells will be critical to our understanding of the biological orchestration that controls neovascularisation in health and disease.

## Conclusion

An increasing number of studies have documented the capacity of EV-bound miRNAs to modulate the angiogenic programs of ECs and to control neovascularisation following MI. EV cargo depends on several factors, including the cell source, the conditions in which EVs have been generated, and the isolation and purification techniques employed. These factors, along with the dose used, may determine the beneficial or detrimental effects of EVs on angiogenesis. EV-based therapeutic approaches, however, are still challenging since our knowledge of the mechanisms of miRNA sorting in EVs, EV release, EV-miRNA uptake and the stoichiometry required for the therapeutic effects of EV-miRNA treatment is limited. A broader understanding of these processes, together with the validation of accurate technologies for the clinical-grade quality control of EVs, would significantly delineate the benefit-risk balance, and open up new opportunities for therapeutic strategies.

## Author Contributions

DK, AB, MB, and AHB outlined concept and overview of review. DK wrote the manuscript. AB, AHB, MB, PdC, and LdW reviewed and edited the manuscript. DK designed and prepared the figures. All authors listed have made a substantial, direct and intellectual contribution to the work, and approved it for publication.

## Conflict of Interest

The authors declare that the research was conducted in the absence of any commercial or financial relationships that could be construed as a potential conflict of interest.
